# 3D Printing-Based Integrated Water Quality Sensing System

**DOI:** 10.3390/s17061336

**Published:** 2017-06-08

**Authors:** Muinul Banna, Kaustav Bera, Ryan Sochol, Liwei Lin, Homayoun Najjaran, Rehan Sadiq, Mina Hoorfar

**Affiliations:** 1 School of Engineering, University of British Columbia, Kelowna, BC V1V 1V7, Canada; banna.mh@gmail.com (M.B.); homayoun.najjaran@ubc.ca (H.N.); Rehan.Sadiq@ubc.ca (R.S.); 2 Indian Institute of Technology, Kharagpur, West Bengal 721302, India; kaustav0bera@gmail.com; 3 Department of Mechanical Engineering, University of Maryland, College Park, MD 20742, USA; rsochol@umd.edu; 4 Department of Mechanical Engineering, University of California Berkeley, Berkeley, CA 94720, USA; lwlin@me.berkeley.edu

**Keywords:** 3D-Printing, miniaturized sensors, water quality, online monitoring

## Abstract

The online and accurate monitoring of drinking water supply networks is critically in demand to rapidly detect the accidental or deliberate contamination of drinking water. At present, miniaturized water quality monitoring sensors developed in the laboratories are usually tested under ambient pressure and steady-state flow conditions; however, in Water Distribution Systems (WDS), both the pressure and the flowrate fluctuate. In this paper, an interface is designed and fabricated using additive manufacturing or 3D printing technology—material extrusion (Trade Name: fused deposition modeling, FDM) and material jetting—to provide a conduit for miniaturized sensors for continuous online water quality monitoring. The interface is designed to meet two main criteria: low pressure at the inlet of the sensors and a low flowrate to minimize the water bled (i.e., leakage), despite varying pressure from WDS. To meet the above criteria, a two-dimensional computational fluid dynamics model was used to optimize the geometry of the channel. The 3D printed interface, with the embedded miniaturized pH and conductivity sensors, was then tested at different temperatures and flowrates. The results show that the response of the pH sensor is independent of the flowrate and temperature. As for the conductivity sensor, the flowrate and temperature affect only the readings at a very low conductivity (4 µS/cm) and high flowrates (30 mL/min), and a very high conductivity (460 µS/cm), respectively.

## 1. Introduction

Online water quality monitoring is becoming an essential part of large water distribution systems (WDS) [1] to ensure that contamination through accidental [2] or deliberate means do not affect the consumers. To maintain drinking water quality throughout the water distribution system, water samples are collected and tested in advanced laboratories by highly-skilled personnel [3]. This type of monitoring restricts the sampling frequency, which in turn increases the probability of disease outbreak. Thus, there is an urgent need for real-time online water quality monitoring systems to be installed throughout WDS. Most urban purification plants have real-time monitoring sensors in the upstream of WDS; however, the placement of online water quality monitoring sensors throughout WDS has not yet been feasible, primarily due to the high cost and low reliability of the sensors [4,5]. Additionally, the pressure at which water is supplied to the customer is much higher than the amount that most sensors can tolerate [6,7]. Miniaturized sensors successfully developed in laboratories can provide a reliable measure of contamination in the water. The performance of the sensors has been examined in laminar flow conditions and at an ambient pressure and temperature [8]. Thus, installing micron-size sensors in WDS requires that the flowrate and pressure that will be encountered by the sensors must be low. Primarily, the water velocity through the sensors must be low enough to prevent the flow from becoming turbulent. Additionally, the water extracted from the system for the purpose of monitoring cannot be redirected back into the system. Therefore, the flowrate at which the water is extracted for sampling must be minimized. The pressure and flowrate at different stages of the WDS also change [6]. Thus, it has to be ensured that the changes in the pressure and flowrate in WDS do not affect the flow through the sensors, which must be kept laminar throughout the sampling process. 

**Figure A1:**
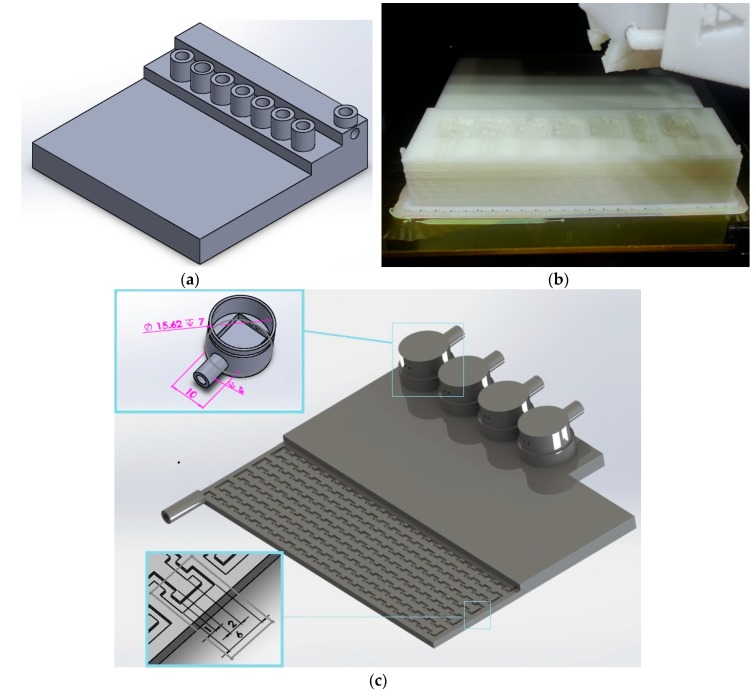

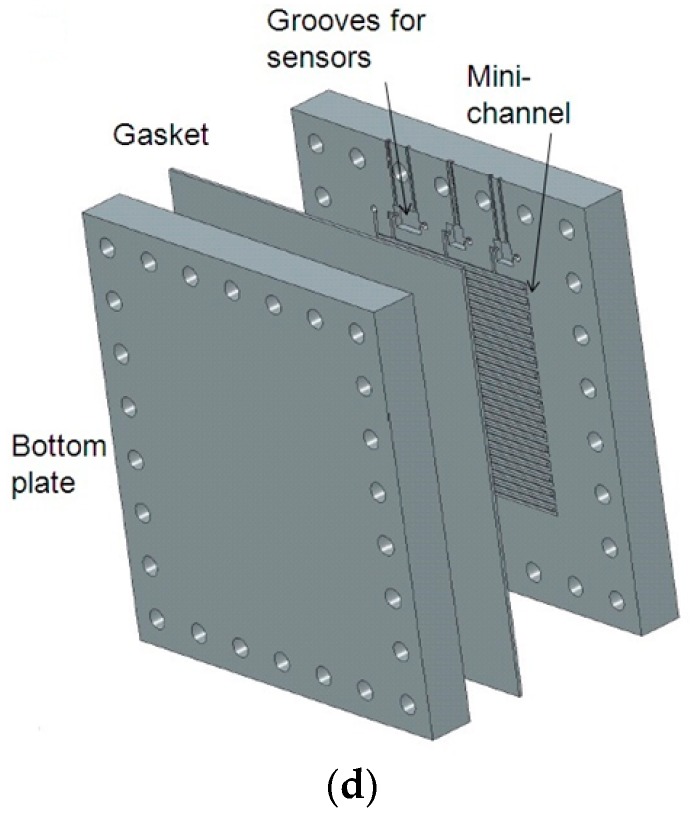
(**a**) Design of the interface; (**b**) Fabrication of the interface using a 3D printer; in this approach, the part was printed on a hop plate and stopped for inserting sensors; (**c**) Dimensions of final 3D print design for reproduction; and (**d**) design of first prototype. Using two polymethyl methacrylate plates with channels machined into it.

In this paper, an interface is designed to meet the conditions required for water quality monitoring using sensors developed recently [8]. Previously, a 2D model was developed to optimize the shape of the channel inside the interface so that the changes in the pressure and flowrate can be minimized [9]. This initial interface was fabricated by machining two polymethyl methacrylate [8] plates attached with screws (Figure A1d). This involved an expensive process of machining, costing more than $300 USD (including the material and fittings) for one interface, which is not a sustainable long-term solution for monitoring numerous nodes in WDS. In addition to the cost, there were two other issues: (i) hermetic sealing was not ensured between the plates; and (ii) for each sensor replacement, the whole interface had to be taken apart, making the long-term maintenance of the device cumbersome. For addressing these issues, here we present a 3D printing approach for the fabrication of the interface. Recently, a variety of 3D printing technologies have been widely used to fabricate components and devices for applications in biochemical sciences [10], microfluidics [11], and biomedical engineering [12] using stereo-lithography [13,14,15], multijet/polyjet modeling [16], and extrusion-based 3D printing technologies (such as material extrusion or fused deposition modeling (FDM)) [17,18]. For example, in biochemical sciences, 3D printing has been used to print organs (e.g., bionic ear, microvascular formation [10]). A mold, used to cast bony in-growth surfaces of the Co-Cr (ASTM F75) alloy, has also been fabricated using 3D printing [12]. Au et al. [13] showed how to 3D print a device for the automated routing, dispensing, mixing, and/or separation of fluids through microchannels using stereo-lithography. A smartphone-based flurometer has also been developed using a 3D printed micro-fluidic chip [19]. Following the high applicability of the 3D printing, the performance of 3D printers/printing processes has also been evaluated in different studies [20,21]. A lot of effort has focused on the optimization of 3D printing process parameters [22]. Salmone et al. [23] showed how to prevent errors in the design phase with the help of additive manufacturing for an environmental monitoring system consisting of the air temperature, relative humidity, air velocity, luminance, and CO_2_ concentration. The housing of the sensor was created by 3D printing [23].

The conventional fabrication of an interface involves machining an open serpentine channel and grooves in a methacrelyte plate and sealing it with the help of another plate with a gasket in between them (Figure A1d). This approach has several problems: (i) the cost is high; (ii) it is impossible to ensure sealing between the plates; (iii) the whole interface has to be dismantled to change the sensors; and (iv) the flowrate through the interface is very high. In order to solve these issues, a few other approaches were taken (Figure A1a–c). The last prototype shown was used for this study. For this prototype, the cost is considerably lower compared to other designs. The serpentine channel is inside a single block, which eliminates the hermetic sealing issue. The design is modular in the sense that if a single sensor is required to be replaced, there is no need to open up the entire device. The flow rate for a 450 kPa pressure difference was reduced from 200 mL/min to 30 mL/min. To show the applicability of the 3D printed interface, two miniaturized sensors developed for the measurement of pH and conductivity [8] are integrated into the device. The performance of the entire integrated system is tested for different flow rates and temperatures.

There are different types of pH sensors [3]. The pH sensor embedded in the interface is a hydrogel-based pH sensor [8]. As the pH of the water changes, the hydrogel layer on the sensor swells/de-swells, resulting in a change in the electrical properties (conductivity and capacitance) of the hydrogel. The pH of the water can also decrease (increase) as the temperature increases (or decreases), without any change in the level of acidity (alkalinity) of the water [24]. Apart from the change in the actual pH value, which is very low for the temperature range studied here (5–20 °C), the change in the temperature has other effects, such as changing the resistance of the hydrogel layer [24]. Thus, it is essential to calibrate the sensor response at different temperatures, as is done for commercially-available pH sensors [25]. 

The functioning of the conductivity sensor embedded in the 3D printed interface is based upon two interdigitated electrodes [8]. The effect of the change of temperature on the response of the conductivity sensor is also studied here. In essence, for a low concentration of solids, the conductivity of the solution increases with the increase of temperature [26]. Thus, the conductance of different samples of water measured at different temperatures cannot be compared if the conductance is not adjusted to a fixed temperature. A temperature co-efficient is usually used to adjust the conductance [27]. The temperature coefficient of conductance is not constant and the factors governing the change of the coefficient are complex. For instance, the coefficient changes with different electrolytes, and with different concentrations and mixtures of electrolytes, and with temperature. The temperature compensation or adjustment can be linear or non-linear, and it can be performed manually or automatically with a processor implemented within the sensor [28]. However, automatic temperature compensation cannot be very accurate as the conductivity also depends on electrolytes.

The paper is organized as follows: first, the design and fabrication of the interface is elaborated. Then, the experimental setup in which the interface and two types of embedded micro-sensors are attached to a temperature control unit is presented. This is followed by a Theory section, illustrating the relationship between temperature and pH and temperature and conductivity. Finally, with the use of the integrated 3D printed system, the performance of the sensors is evaluated for different temperatures and flowrates. The experimental results are compared against the theoretical values. This comparison is used to compensate for the effect of the temperature on the response of these sensors.

## 2. Interface Fabrication and Testing

**Figure 1:**
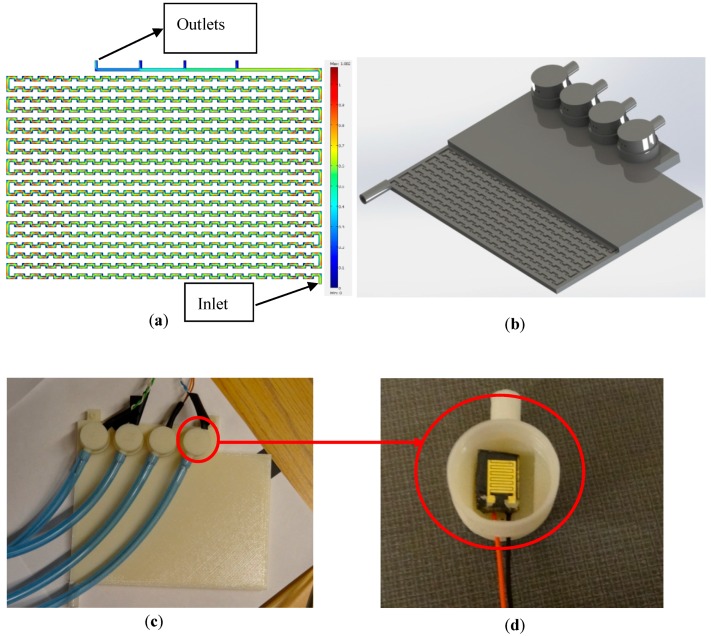
Fabrication of the interface using 3D. (**a**) Comsol simulation of the interface; (**b**) CAD model of the interface; (**c**) fabricated interface; and (**d**) pH sensor fitted into the interface.

A 2D model [29] was used to simulate the interface. COMSOL multi-physics software was used for modeling and simulation purposes (see Figure 1a) [8]. In a recent study, the sensors were tested at different flowrates (as high as 30 mL/min) and the inlet pressure of 450 kPa (presenting the maximum pressure at WDS) [8]. In the previous study [8], such an interface was machined out of Polymethyl methacrylate, which resulted in several issues related to leakage and cost. To overcome these, 3D printing technology was used to reduce the cost, eliminate the leakage problem, and reduce the fabrication time. For this purpose, two different approaches were tested to find the optimum design and process. Figure A1a,b shows the first trial with the purpose of integrating the sensors into the interface during the printing process. In essence, the printing process was stopped in the middle of interface printing to insert the sensors. Since the prototype was printed on top of a heated plate, stopping the process to insert the sensors caused the printed structure to cool down, resulting in misalignment after the printing process was started again. Moreover, once the sensors were assembled into the device, it was not possible to replace them (in case of sensor failure), in which case the interface had to be discarded. Therefore, a new modular interface was designed and fabricated (see Figure 1b,c).

A commercially available desktop 3D design printer (Mojo 3D Printer, TRIMECH, Glen Allen, VA, USA) was used. The printer uses FDM^®^ (Fused Deposition Modeling™) technology developed by Stratasys Inc. (Eden Prairie, MN, USA). Specifically, the printer has a 12.7 cm^3^ working stage with a deposition layer thickness of 0.17 mm and 2D spatial resolution of 0.07 mm. For the model presented here, a P430 ABSplus (ivory) Mojo QuickPack Print Engine [30] was used. For the void space inside the structure, a support material was used. This material was removed by dissolving it with a proprietary solvent provided with the printer (Soluble Support Technology was used to dissolve the support material in a water-based solution). The completed model along with the supporting material was placed in the WaveWash 55 Support Cleaning System (Stratasys Inc., Eden Prairie, MN, USA) along with an Ecoworks tablet (Stratasys Inc., Eden Prairie, MN, USA) and DI Water. The washing unit maintains the right solution temperature and agitation for efficient support removal, with no requirement for additional plumbing. The whole body of the 3D printed part did not have any infill as the structure was very thin (2 mm). The target of not using infill was to ensure that water does not leak through the top or bottom of the interface. The orientations of the 3D printed parts are as shown in Figure A1c. The orientation of the removable module is shown in the exploded view. These orientations were selected to minimize the amount of support material. The final fabrication time was about 4 h, followed by 12 h of washing to remove the support material. The serpentine channel with a very narrow cross sectional area (1 mm × 1 mm) required additional time for cleaning (more than 6 h recommended). Extra efforts were spent on removing the support material from the serpentine channel. The prototype was submerged into the solution for dissolving the support material overnight. Then, the solution was pushed through the serpentine channel with the help of a syringe. The solution was taken out by the syringe after a few minutes. The process of cleaning the serpentine channel interface was repeated until the flow through the interface was achieved. The approximate cost of fabrication by the printer used was around $50 CAD. The time taken for post processing was long (approximately 15 h). However, the presence of an individual was only required during the cleaning of the serpentine channel. Cleaning the serpentine channel required about 3 h, which adds to the cost of fabrication.

## 3. Experimental Setup

**Figure A2:**
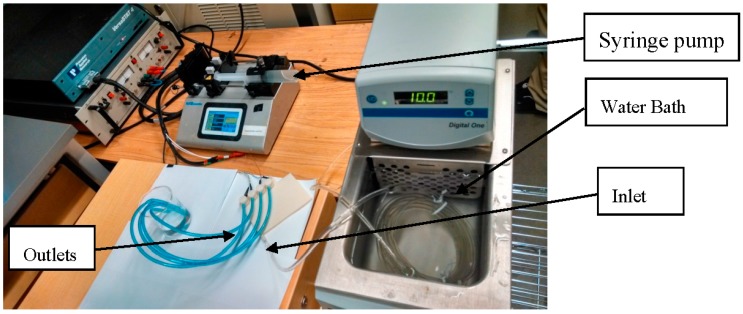
Experimental setup.

The pH and conductivity sensors were successfully integrated into the interface (see Figure 1d). The interface can provide an appropriate flowrate at any pressure ranging from 50 to 450 kPa. Figure A2 shows the experimental setup. The water sample is kept inside a syringe and a coil of plastic tube attached to the syringe. The coil of the plastic tube is kept in the water bath and the other end of the coil is attached to the inlet of the interface. The water from the outlet of the interface is collected into a beaker. The experiments were conducted in three steps: (a) modeling and fabrication of the pressure reducing interface, (b) testing the sensors under static and dynamic conditions, and (c) testing of the sensors at varying temperatures. 

The design for the pressure reducing interface was conceptualized and simulated using COMSOL Multi Physics 3.4 (as explained in Section 2). The final design of the prototype and the necessary adaptors to fix the sensors in their respective positions in the interface was created using SolidWorks2010 (Waltham, MA, USA) (see Figure 1b). Three of the four slots of the sensors were used to house three pH or conductivity sensors (for replication) and the fourth slot held the temperature senor. The interface is responsible for the reduction of the water pressure to a level suitable for the sensors, while allowing the continuous exposure of the sensors to water of the WDS. This integrated system can hence be integrated directly into the pipelines in the water distribution system (WDS). The very low exit flowrate also ensures negligible water bleeding from the pipeline, reducing chances of contamination and/or pressure loss in the distribution system.

Once the sensors were successfully incorporated into the interface, they were tested under both dynamic and static conditions. A series of static experiments were conducted while the sensors were independently immersed in a beaker (50 mL), as well as integrated into the printed interface. For both cases, the water sample and sensors (placed either in the beaker or interface) were immersed in the water bath. No significant difference was observed among the two sets of static measurements. For testing the interface and the sensors under the dynamic condition, the system was connected to a syringe pump (KDS LEGAT0270, manufacturer: kdScientific, Holliston, MA, USA) using a coil of long polyurethane tubing (ID and OD 4.65 mm and 6.3 mm, respectively). The syringe pump was able to supply water at a maximum flowrate of 30 mL/min. This flowrate is almost equal to the simulated flowrate obtained from the model (see Figure 1a). The coil was immersed in a digitally controlled water bath (NesLaB RTE 7, Thermo Scientific, Mississauga, ON, Canada). Finally, the outlet water was collected in a beaker to test for any change in the characteristics of the water inside the interface (see Figure A2). 

In addition to the change in the flowrate (static versus dynamic), the integrated system (including the interface and sensors) was tested at different temperatures (5 °C, 10 °C, 20 °C). To ensure that the temperature set for the water bath was the actual temperature of the water sample that the sensors were exposed to, a miniaturized thermistor (calibrated for a wide range of temperatures) was also integrated in the 3D printed interface. The readings from the thermistor and the water bath were consistent, except for the lowest temperature range and dynamic condition. Therefore, for all the temperatures reported in the results, the readings from the thermistor were used. 

To examine the performance of the integrated system, the sensors were tested against a wide range of pH and conductivity values. For this purpose, known standard buffer solutions of pH 6.4, 7, 8, and 9 were prepared using buffer capsules (pHydrion Buffers manufactured by Micro Essential (Brooklyn, NY, USA). Water samples at different conductivities were prepared by dissolving varying amounts of calcium chloride (CaCl_2_) (analytical grade by Sigma Aldrich, Oakville, ON, Canada) in DI water. The sensors were connected to a potentiostat (Versa STAT 4 by Princeton Applied Research, Oak Ridge, TN, USA) to obtain the response of the sensors. Each experiment was repeated three times to ensure the reproducibility of the measurements.

## 4. Theory

It is evident from the literature that the conductivity or the pH [31] of water changes with the change of temperature. The conductivity measurements obtained at temperatures other than 18 °C are corrected by using a temperature coefficient, which compensates for the change of conductance per each degree of temperature change. This coefficient is frequently considered as c = 0.02, i.e., approximately 2% per each degree of temperature change in the case of dilute aqueous solutions [26]. More recently, c = 0.025 [26] was used by aquatic biologists for temperature adjustments using the following relation:

(1) $$K_{18}=\frac{1000000}{R_{t}\left[1+c\left(t-18\right)\right]}$$

where, $K_{18}$ represents conductance (µS) at 18 °C, t is the temperature at which the measurement is obtained, $R_{t}$ presents the resistance at the temperature t, and c  epresents the temperature coefficient. If $K_{18}$ can be represented in terms of resistance, one can express the temperature coefficient (c) by the following equation:

(2) $$c=\frac{R_{18}-R_{t}}{R_{t}\left(t-18\right)}$$

This coefficient, which compensates for the change in pH with the change of temperature, is called the solution temperature coefficient (pH/°C): if a solution has a temperature coefficient of −0.045 pH/°C, it means that the pH reduces by 0.045 units for every degree increase in temperature. The coefficient, in most cases, has to be determined empirically [24].

## 5. Results

**Figure A3:**
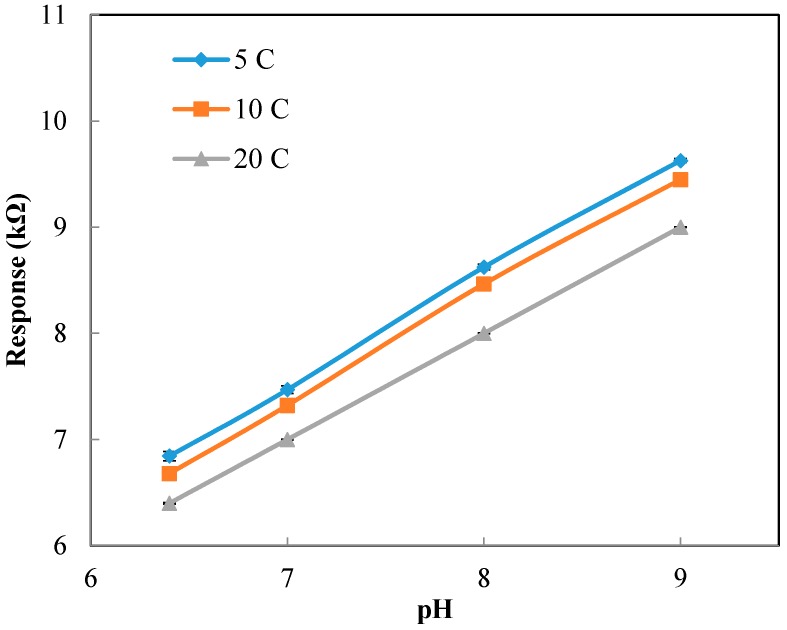
Calibration curve for the pH sensor at the static condition for different temperatures.

**Figure A4:**
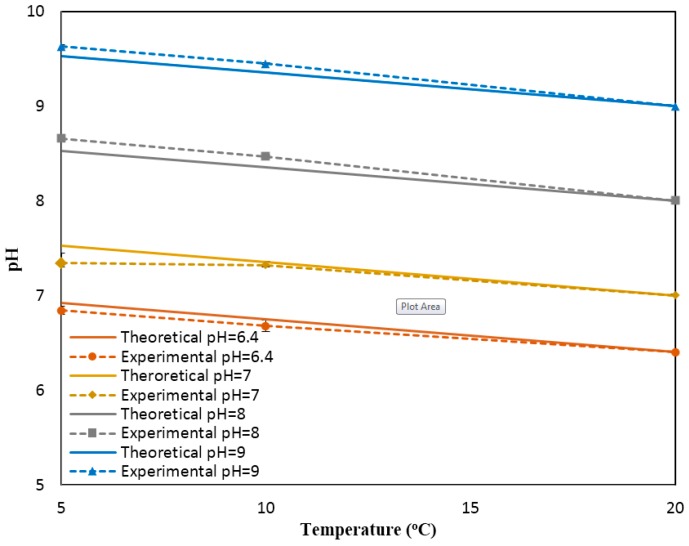
Effect of temperature on the response of the pH sensor compared to the theoretical values (both experimental and theoretical values were obtained at the static condition).

Figure A3 shows the calibration curve for the pH sensor implemented in the 3D printed interface. The calibration curve was used to calculate the pH values from the response (kΩ) of the sensor measured at the static condition. Based on the calibration curve, the pH values were determined from the sensor response measured at different temperatures (but still at the static condition). Figure A4a presents the experimental pH values compared against the theoretical values obtained based on the linear relationship between pH and temperature (pH value reduces 0.035 units for 1 °C increase in temperature). The results show a great agreement between the experimental pH values (measured at different temperatures and the static condition) and theoretical values. The results presented include the average and standard deviation between the three replications conducted. Since the errors are very small, they are not apparent in the figure. In summary, we can conclude that temperature has a considerable effect on the response of the sensor. 

**Figure 2:**
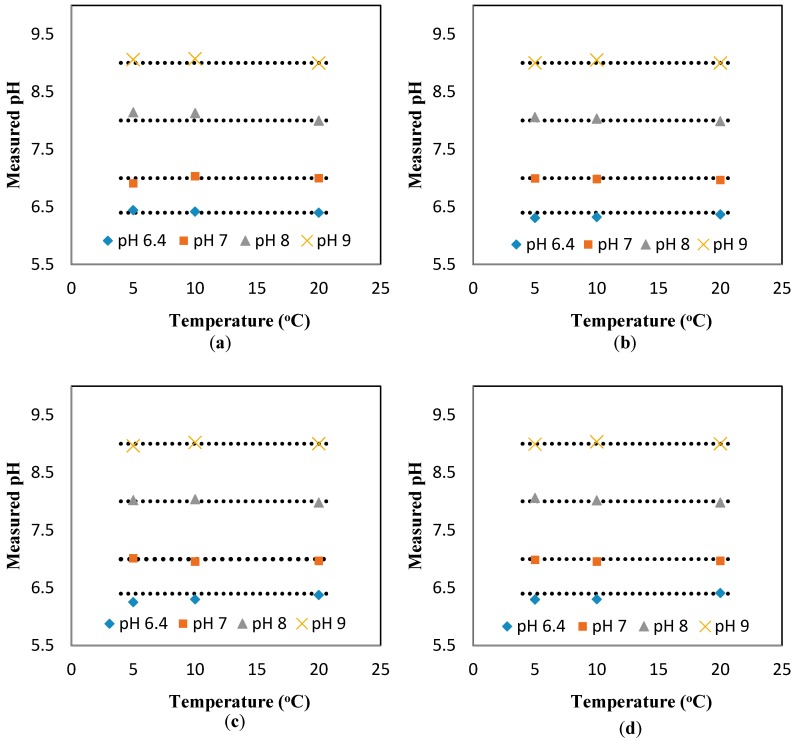
The measured pH after adding the compensation factor (a function of measured pH and temperature) at flowrate (**a**) 0 mL/min; (**b**) 10 mL/min; (**c**) 20 mL/min; and (**d**) 30 mL/min.

**Figure A5:**
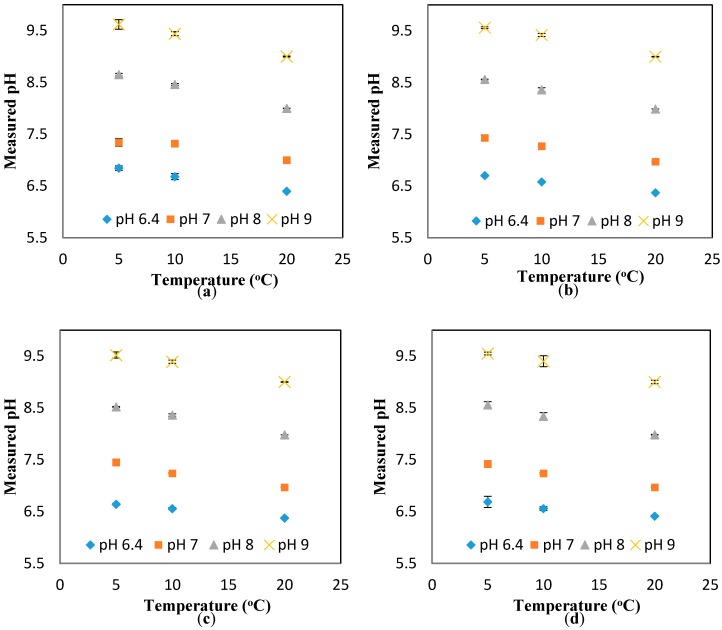
Effect of temperature on the response of the sensors for different pH solution at the flowrates of (**a**) 0 mL/min; (**b**) 10 mL/min; (**c**) 20 mL/min; and (**d**) 30 mL/min.

To study the effect of the flowrate on the response of the sensor, the same experiments were repeated for the dynamic condition at three flowrates (10, 20, 30 mL/min). Figure A5b–d shows the changes in the pH values as a function of the temperature for different flowrates. It is apparent that regardless of the flowrate, the linear relationship between temperature and pH is still valid (for example, the average slope of temperate vs. measured pH is −0.037 ± 0.0014 for the flowrates of 10, 20, and 30 mL/min). Thus, the response of the sensor can readily be compensated for to remove the effect of temperature and report the pH value at the room temperature. The relationship used for such a compensation is as follows: ${\mathrm{pH}}_{c}={\mathrm{pH}}_{m}\left[1+0.0039\left(t-20\right)\right]$, where ${\mathrm{pH}}_{m}$ and ${\mathrm{pH}}_{c}$ represent the measured pH at temperature t and the compensated pH at 20 °C, respectively. This relationship was applied to the results presented in Figure A5 to find the compensated value of pH at different flowrates, as shown in Figure 2a–d. The deviation from the flat line in these figures shows the error of the sensor, which is less than ±0.15. 

**Figure 3:**
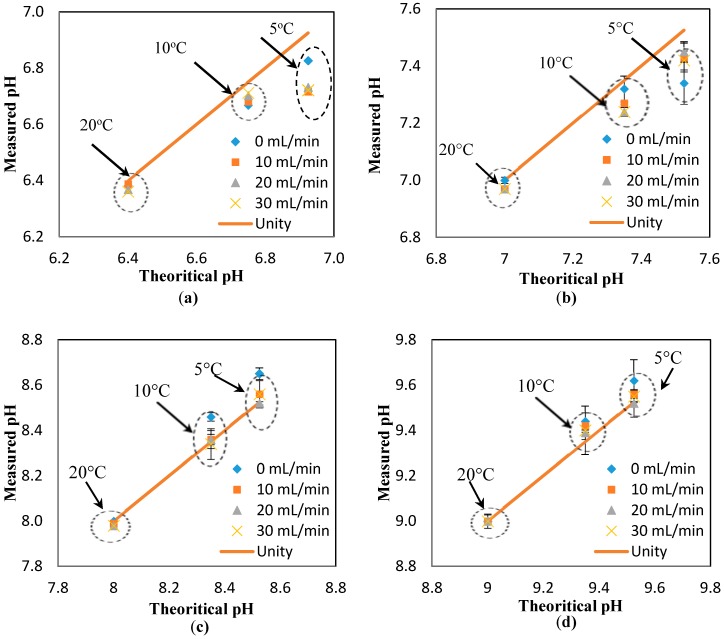
Effect of the flowrate on the response of the sensor measured at different temperatures and dynamic condition. The curves illustrate the deviation from the theoretical pH values (using the “unity line”) for different pH ranges of (**a**) 6.4; (**b**) 7; (**c**) 8; and (**d**) 9.

The results in Figure A5 also suggest a minimal effect of the flowrate on the response of the pH sensors. To further investigate this suggestion, the pH results measured at different temperatures (i.e., 5, 10, and 20 °C) and different flowrates are presented against the theoretical value (obtained at the static condition). The “unity line” is used to show the deviation of the measured values (at different flowrates) from the theoretical values (estimated at different temperatures, but the static condition). Thus, the closer the points to the unity line, the lower the effect of the parameter studied here (i.e., flowrate). Based on the results presented in Figure 3a–d, it is safe to conclude that the effect of the flowrate on the response of the pH sensor is minimal, even at the different pH ranges studied here (i.e., 6.4, 7, 8, 9). In fact, the maximum deviation from the theoretical value is 1.4%, which is in the range of the accuracy of the pH sensor developed here [8].

**Figure A6:**
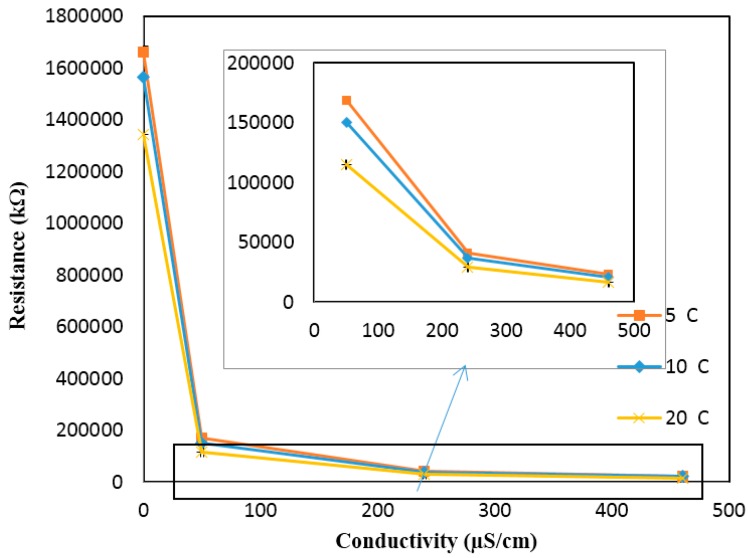
Calibration curve for the conductivity sensor. The inset shows the response of the sensors at high conductivity.

**Figure A7:**
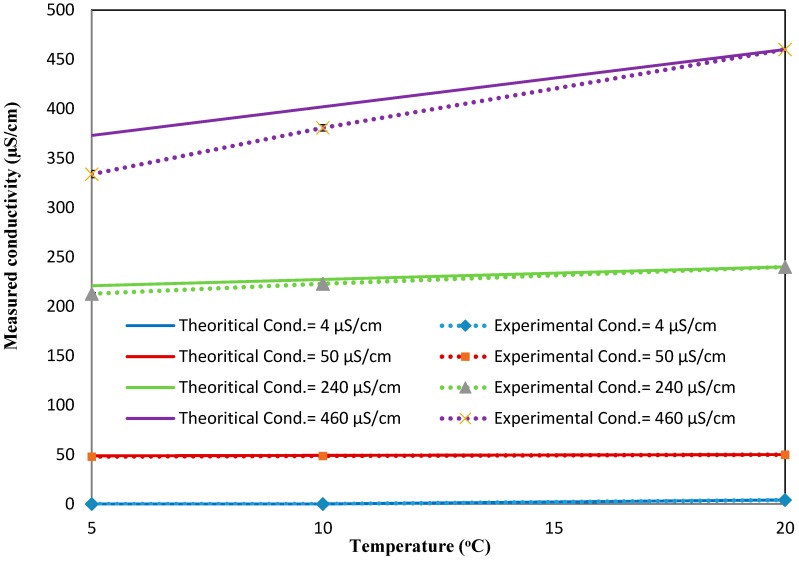
Effect of temperature on the response of the conductivity sensor compared to the theoretical values (both experimental and theoretical values were obtained at the static condition).

Figure A6 shows the calibration curve for the conductivity sensor implemented in the 3D printed interface. The calibration curve was used to calculate the conductivity values from the response (kΩ) of the sensor measured at the static condition. The response of the sensor for the high conductivity solutions is enlarged, as it is very low compared to the response obtained for the low conductivity solutions. Based on the calibration curve, the conductivity values were then determined from the sensor’s response measured at different temperatures (but still at the static condition) and compared against the theoretical values (see Figure A7). The results presented in Figure A7 include the average and standard deviation between the three replications conducted at the static condition for different temperatures. Since the errors are very small, they are not apparent in the figure. It is evident from the figure that the measured conductivity value increases (or the sensor response decreases) by increasing the temperature of a solution of a known conductivity. The increase in the conductivity values is in the range of 1.5% to 2% for every degree centigrade temperature increase. This is in agreement with the theoretical values (see the coefficient c in Section 4).

**Figure 4:**
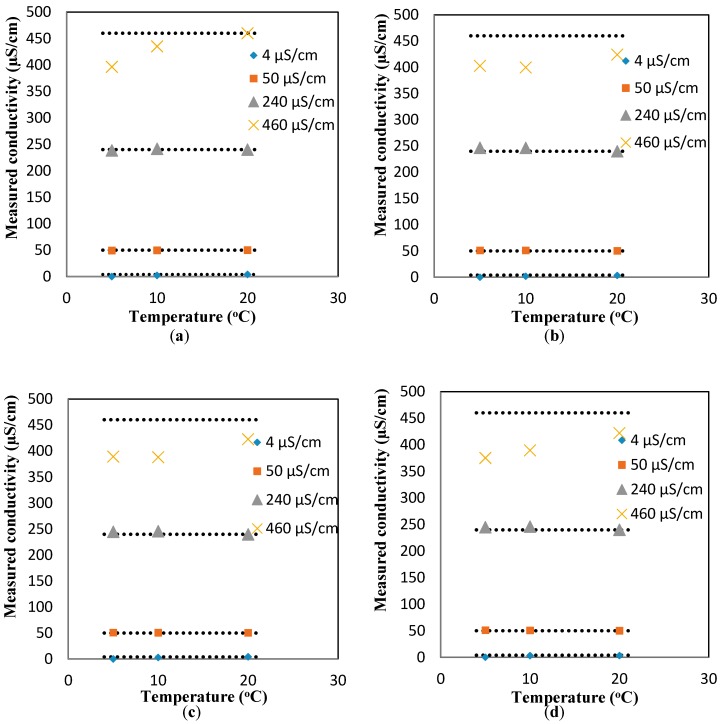
The measured conductivity after adding the compensation factor (a function of measured conductivity and temperature) for different conductivity solutions at flowrates of (**a**) 0 mL/min; (**b**) 10 mL/min; (**c**) 20 mL/min; and (**d**) 30 mL/min.

**Figure A8:**
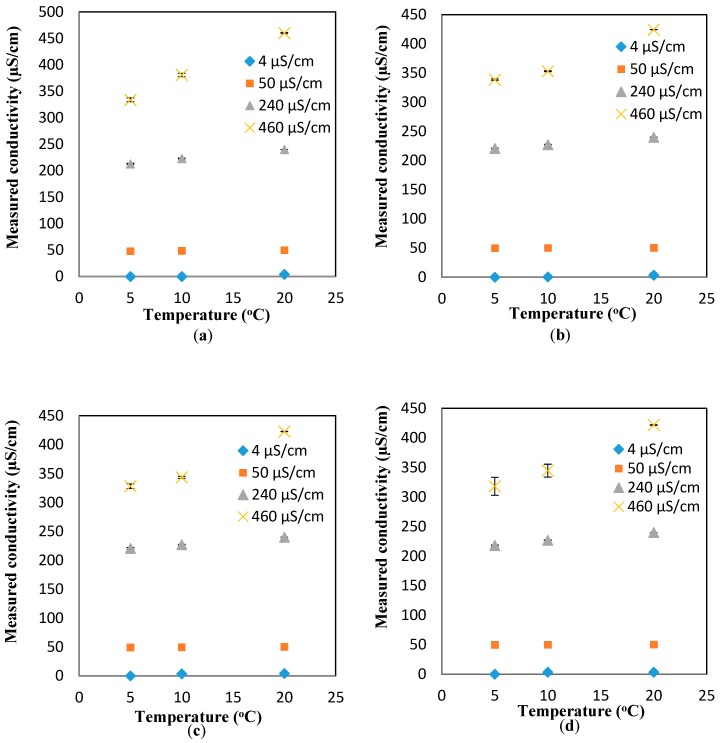
Effect of temperature on the response of the conductivity sensor for different conductivity solutions at the flowrates of (**a**) 0 mL/min; (**b**) 10 mL/min; (**c**) 20 mL/min; and (**d**) 30 mL/min.

To study the effect of the flowrates on the response of the conductivity sensor, the same experiments were repeated for the dynamic condition at three flowrates (10, 20, and 30 mL/min). Figure A8b–d show the changes in the conductivity values as a function of the temperature for different flowrates. It is apparent that regardless of the flowrate, the linear increasing trend between the temperature and conductivity is still valid. However, the average slopes of the conductivity-vs-temperature curves for all the conductivity values (4, 50, 240, 460 µS/cm) are smaller than those measured in the static conditions (i.e., the average slopes for the conductivity-vs-temperature curves for all the flowrates at 4, 50, 240, 460 µS/cm are 0.241 ± 0.038, 0.0668 ± 0.0.0468, 1.448 ± 0.241, and 6.948 ± 1.031, respectively). These average slopes correspond to the coefficients of c of 0.7%, 1.0%, 1.2%, and 1.5%, respectively, which are smaller than the theoretical value mentioned in the Theory Section. Similar to pH, a compensation relationship for conductivity is added to the measured responses to obtain the comparable conductivity values at 20 °C. The relationship used for such a compensation is as follows: $k_{c}=k_{m}-0.000375\times \left(t-20\right)k_{m}^{2}$, where $k_{m}$ and $k_{c}$ are the measured conductivity at temperature t and the the compensated conductivity at 20 °C, respectively. This relationship was applied to the results presented in Figure A8 to find the compensated values of conductivity at different flowrates, as shown in Figure 4a–d. The deviation from the flat line shows the error of the sensor. For low conductivity solutions, the error is minimal (less than 3.5%). However, the compensated conductivity values deviate considerably (18%) from the flat line for the largest highest conductivity range tested here (i.e., 460 µS/cm).

**Figure 5:**
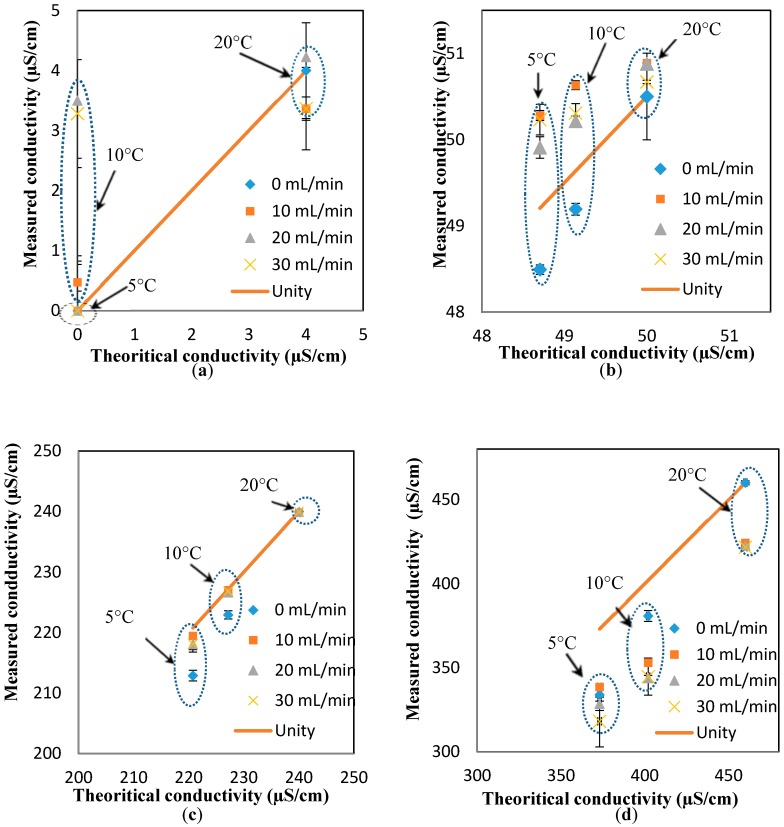
Effect of temperature on the response of the conductivity sensor for different conductivity solutions at flowrates of (**a**) 0 mL/min; (**b**) 10 mL/min; (**c**) 20 mL/min; and (**d**) 30 mL/min.

To investigate the effect of the flowrates on the response of the conductivity sensor, the “unity line” approach (similar to the pH results) is used to show the deviation of the measured values (at different flowrates) from the theoretical values (estimated at different temperatures, but the static condition). In essence, the closer the points to the unity line, the lower the effect of the parameter studied here (i.e., flowrate). Based on the results presented in Figure 5a–d, it is safe to conclude that the effect of the flowrate on the response of the pH sensor is minimal for most of the conductivity values and flowrates, expect for the measurements at low conductivity and high flowrates (4 µS/cm and 30 mL/min, respectively).

## 6. Conclusions

An interface was fabricated using 3D printing technology to reduce the pressure of the water mains so that the micro-sensors developed here can be used for online water quality monitoring. Based on different 3D printing procedures, the optimum geometry and approach were identified to fabricate a modular structure, allowing for integrating each of the sensors with appropriate adaptors. Such a modular arrangement allows for the independent handling and replacement of each sensor, without hampering the functionality of the other components. The performance of the system was tested by integrating the pH and conductivity sensors and testing at different temperatures and flowrates. Commercial sensors use a specific temperature correction method to compensate for the fluctuation in the system’s reading. For this purpose, a temperature sensor was also integrated to simultaneously measure the temperature, along with the pH and conductivity. The response of the sensors in such an interface was compared to the theoretical values. The results show that the reading from the pH sensors matches the theoretical values for all the ranges of flowrates and temperatures tested here. The same conclusion was obtained for the response of the conductivity sensor for the majority of the conditions, except for low conductivity (4 µS/cm) and a high flowrate (30 mL/min). Similar to commercially available sensors, compensation factors were implemented to eliminate the effect of temperature on the readings of the sensors. The results show that temperature has a minimal effect on the readings of both types of sensors, expect for samples with very high conductivity (460 µS/cm).

## References

[1] Craun G., Brunkard J., Yoder J., Roberts V., Carpenter J., Wade T., Calderon R., Roberts J., Beach M., Roy S. (2010). Causes of Outbreaks Associated with Drinking Water in the United States from 1971 to 2006. Clin. Microbiol. Rev..

[2] Angulo F., Tippen S., Sharp D., Payne B., Collier C., Hill J., Barrett T., Clark R., Geldreich E., Donnell H. (1997). A community waterborne outbreak of salmonellosis and the effectiveness of boil water order. Am. J. Public Health.

[3] Banna M.H., Imran S., Francisque A., Najjaran H., Sadiq R., Rodriguez M., Hoorfar M. (2014). Online drinking water quality monitoring: Review on available and emerging technologies. Crit. Rev. Environ. Sci. Technol..

[4] O’Halloran R., Fogelman S., Zhao H. (2009). Urban water security research alliance technical report. Current Online Water Quality Monitoring Methods and Their Suitability for the Western Corridor Purified Recycled Water Scheme.

[5] Shastri Y., Diwekar U. (2006). Sensor Placement in Water Networks: A Stochastic Programming Approach. J. Water Res. Plan. Manag..

[6] Kelowna Joint Water Committee, 2012 Kelowna Integrated Water Supply Plan. http://apps.kelowna.ca/CityPage/Docs/PDFs/Council/Meetings/Council%20Meetings%202012/2012-11-05/Item%20(AM)%204.2%20-%20Kelowna%20Integrated%20Water%20Supply%20Plan.pdf.

[7] Hach Company, pHD sc Digital Differential pH/ORP Sensors User Manual. http://www.hach.com/asset-get.download.jsa?id=7639983075.

[8] Banna M.H., Najjaran H., Sadiq R., Imran S., Rodriguez M., Hoorfar M. (2014). Miniaturized water quality monitoring pH and conductivity sensors. Sen. Actuators B Chem..

[9] Banna M.H., Imran S., Najjaran H., Sadiq R., Rodriguez M., Hoorfar M. Simulation of constant pressure and flowrate through mini-channels inserted into distribution systems (WDS). Proceedings of the ASME 2012 10th International Conference on Nanochannels, Microchannels, and Minichannels (ICNMM2012).

[10] Gross B.C., Erkal J.L., Lockwood S.Y., Chen C., Spence D.M. (2014). Evaluation of 3D Printing and Its Potential Impact on Biotechnology and the Chemical Sciences. Anal. Chem..

[11] Erkal J.L., Selimovic A., Gross B.C., Lockwood S.Y., Walton E.L., McNamara S., Martinb R.S., Spence D.M. (2014). 3D printed microfluidic devices with integrated versatile and reusable electrodes. Lab Chip.

[12] Curodeau A., Sachs E., Caldarise S. (2000). Design and fabrication of cast orthopedic implants with freeform surface textures from 3-D printed ceramic shell. J. Biol. Mater. Res..

[13] Au A., Bhattacharjee N., Horowitz L.F., Chang T.C., Folch A. (2015). 3D-printed microfluidic automation. Lab Chip.

[14] Bhargava K., Thompson B., Malmstadt N. (2014). Discrete elements for 3D microfluidics. Proc. Natl. Acad. Sci..

[15] Au A., Lee W., Folch A. (2014). Mail-order microfluidics: Evaluation of stereolithography for the production of microfluidic devices. Lab Chip.

[16] Sochol R.D., Sweet E., Glick C.C., Venkatesh S., Avetisyan A., Ekman K.F. (2016). 3D printed microfluidic circuitry via multijet-based additive manufacturing. Lab Chip.

[17] Sochol R.D., Gupta N.R., Bonventre J.V. (2016). A Role for 3D Printing in Kidney-on-a-Chip Platforms. Curr. Transplant. Rep..

[18] ISO/ASTM52900—15 Standard Terminology for Additive Manufacturing.

[19] Hossain A., Canning J., Ast S., Rutledge P.J. (2015). Lab-in-a-Phone: Smartphone-Based Portable Flurometer for pH Measurements of Environmental Water. IEEE Sens. J..

[20] Yang H., Lim J., Liu Y., Qi X., Yap Y. (2017). Performance evaluation of pro-jet multi-material jetting 3D printer. Virtual Phys. Prototyp..

[21] Lee J., Zhang M., Yeong W. (2016). Characterization and evaluation of 3D printed microfluidic chip for cell processing. Microfluid. Nanofluid..

[22] Salmi M., Ituarte I.F., Chekurov S., Huotilainen E. (2016). Effect of build orientation in 3D printing production for material extrusion, material jetting, binder jetting, sheet object lamination, vat photopolymerisation, and powder bed fusion. Int. J. Collab. Enterp..

[23] Salamone F., Danza L., Meroni I., Pollastro M. (2017). A Low-Cost Environmental Monitoring System: How to Prevent Systematic Errors in the Design Phase through the Combined Use of Additive Manufacturing and Thermographic Techniques. Sensors.

[24] Barron J., Ashton C., Geary L. The Effects of Temperature on pH Measurement. http://reagecon.com/pdf/technicalpapers/Effects_of_Temperature_on_pH_v4-_TSP-01–2.pdf.

[25] James R., Dindal A., Willenberg Z., Riggs K. (2005). Analytical Technology, Inc. Q45wq Continuous Multiparameter Water Quality Monitor. Environmental Technology Verification Report.

[26] Smith S.H. (1962). Temperature correction in conductivity measurements. Limnol. Oceanogr..

[27] Thermo Scientific^TM^, Orion^TM^ Laboratory Products Catalog. https://tools.thermofisher.com/content/sfs/brochures/ThermoScientific-Orion-Labratory-Catalog.pdf.

[28] Barron J., Ashton C. The Effect of Temperature on Conductivity Measurement. http://reagecon.com/pdf/technicalpapers/Effect_of_Temperature_TSP-07_Issue3.pdf.

[29] Banna M.H., Najjaran H., Sadiq R., Rodriguez M., Imran S., Hoorfar M. (2014). Fabrication and Testing of a Miniaturised Water Quality Monitoring pH and Conductivity Sensors. Nanotech.

[30] Gausemeier J., Echterhoff N., Wall M. (2013). Thinking Ahead the Future of Additive Manufacturing—Innovation Road-Mapping of Required Advancements.

[31] Galster H. (1991). pH Measurement: Fundamentals, Methods, Applications, Instrumentation.

